# “We lost a lot, but something good came out of it too:” Exploring the impact of the COVID-19 pandemic on the mental wellbeing of British Muslim Pakistani women with family responsibilities

**DOI:** 10.1371/journal.pone.0292614

**Published:** 2023-10-05

**Authors:** Halima Iqbal, Bridget Lockyer, Syka Iqbal, Josie Dickerson

**Affiliations:** 1 Faculty of Health Studies, University of Bradford, Bradford, United Kingdom; 2 Bradford Institute for Health Research, Bradford Teaching Hospital NHS Foundation Trust, Bradford, United Kingdom; Federal Ministry of Agriculture and Rural Development, NIGERIA

## Abstract

**Background:**

The COVID-19 pandemic and associated restrictions caused major disruption globally, shedding light on the unprecedented strain upon the mental health and wellbeing of individuals around the world. Poor mental health in the pandemic is reported to be greater in women, with mothers being at increased risk. It is unclear whether there are differences in the impact of mental wellbeing on some ethnic groups over others. The aim of this study was to explore the experiences of British Muslim Pakistani women with family responsibilities during the COVID-19 pandemic, two years on from the first lockdown.

**Methods:**

Qualitative interviews with women were conducted via telephone using a semi-structured topic guide. The sample included 25 British Muslim Pakistani women with family responsibilities, both English and non-English speaking. Women lived in households that ranged in number and included extended family. Key themes were determined using thematic analysis.

**Results:**

Results were grouped under three themes. These were (1) Community, cultural and religious contributors to poor mental wellbeing, (2) religious and cultural mediators of mental distress, and (3) perceived positive impact on lifestyle. British Muslim Pakistani women were psychologically distressed by the high rates of virus transmission and deaths in their communities and at the prospect of older members of their extended family developing the virus. The impact of restrictions on fundamental religious and cultural interactions further exacerbated poor mental wellbeing in this population. Religion, community social capital and larger household structures were all effective coping strategies for British Muslim Pakistani women. Positive impacts of the pandemic included becoming closer to family and faith, and increased work/life harmony.

**Conclusions:**

An exploration of religious and cultural coping mechanisms should be used to inform future national pandemic preparedness plans, as well as effective strategies for building and maintaining social capital. This may increase adherence to physical distancing and other protective behaviours in populations.

## Background

The COVID-19 pandemic has disrupted many facets of life, resulting in social and economic distress on unprecedented levels. Social distancing, isolation, and country-wide lockdown measures aimed at reducing virus transmission have created stressful experiences for families, particularly those who were already vulnerable [1]. Many ethnic minority groups have disproportionately experienced the negative effects of the COVID-19 pandemic compared to the wider British population [2] having been impacted by both the high virus transmission and socio-economic consequences [3]. These differences in outcomes have been attributed to factors such as working in front-line occupations; living in large, multigenerational households; prevalence of pre-existing health conditions; discrimination, and a lack of access to health or community services [4–6].

There is a plethora of evidence from the United Kingdom demonstrating that lockdown measures to control the spread of the COVID-19 virus have led to an increase in poor mental health [7–9]. There is also evidence that mental health inequalities have been compounded by the pandemic with the groups suffering from the poorest mental health pre-pandemic also having the largest deterioration [10]. Other factors that have been found to be associated with a deterioration in mental health are low socioeconomic status, loneliness, being an adult, a woman and being a parent to a young child [9–11]. Evidence of the impact of the pandemic on the mental health of ethnic minority groups has been unclear. Two recent studies that have included a large proportion of ethnic minority mothers from Pakistani, Bangladeshi, and White other backgrounds have reported no differences in the likelihood of experiencing mental ill health during the pandemic once other key risk factors have been controlled for [11, 12]. These studies instead showed that the key risks associated with increased mental ill health during the pandemic were financial and food insecurity, loneliness, low levels of physical activity and a poor partner relationship. There was also a greater prevalence of depression in those families living in areas of London compared to Bradford.

One of these studies (Dickerson et al. 2022) looked at the likelihood of experiencing depression during, compared to before, the pandemic and reported differences in the magnitude of the risks associated with becoming depressed by ethnicity: Pakistani heritage mothers were more likely to experience depression if they were lonely or had a poor partner relationship, but were far less likely to experience depression if they lived in a large household; whereas White British women were more likely to experience depression if they were financially insecure or had low levels of physical activity [11]. The authors noted that more exploratory research in this area would enable accurate understanding of the possible differing experiences and needs of this population.

Stress factors for Pakistani women have been reported to stem from their South Asian cultural background including their cultural, gender role as women in the family and cultural conflict concerning izzat (i.e., honour/respect), social isolation and migration related stress [13–15]. There may also be some protective factors at play in this community such as living in a close-knit community and multi-generational households that may protect against risk factors such as loneliness and financial insecurity. It is important to note, however, that both aforementioned studies [11, 12] used mainly quantitative approaches and were therefore unable to explore in detail, some of the experiences of British Pakistani women during the pandemic to gain a richer understanding of the factors that may have contributed to their mental wellbeing. This study, therefore, uses qualitative interviews to understand in-depth the mental health experiences, including risk and protective factors of British Muslim Pakistani women with family responsibilities throughout the pandemic in Bradford, UK. This location was selected due to the exceptionally high rates of virus transmission and mortality from COVID-19 in the region. As a consequence of these high rates, there were tighter regional restrictions in Bradford, as well as in the more deprived cities of Northern England through 2020–2021 [11]. As previously mentioned, being a woman, and a parent to a young child, was evidenced to exacerbate poor mental health during the pandemic. As such, Pakistani women with family responsibilities more specifically, were selected due to their unique role in family dynamics and potential vulnerabilities during the pandemic.

## Methods

### Study design and setting

Bradford is the 6^th^ largest city in the UK, located in the North of England. The city has a multi-ethnic population in excess of 500,000 people and has some of the highest levels of deprivation and poor health outcomes in England [16]. There is a well-established South Asian presence in the city and close to half of the South Asian population in Bradford is of Pakistani, Mirpuri origin, and this constitutes a three-generation community [16]. The Pakistani community tend to live in areas where they are the majority ethnic group [16]. Throughout the pandemic, Born in Bradford (BiB), utilised its vast infrastructure of community and stakeholder relations and birth cohort studies [16–18] to understand the short, medium and long term societal impacts of the COVID-19 response on health trajectories and inequalities. The BiB COVID-19 research study included three longitudinal surveys throughout the pandemic, and responsive and reflexive in-depth qualitative studies to explore in more detail issues identified by the community and stakeholders and/or through the survey findings. The present study presents findings from a qualitative study undertaken as a part of this COVID-19 research programme.

### Ethical approval

National Health Service (NHS) Ethical approval had already been granted for the BiB COVID-19 study (HRA and Bradford/Leeds ethics committee (BiB Growing Up study 16/YH/0320; BiBBS study 12/YH/0455) and an amendment was approved for the current qualitative study (Ref no: 207543).

### Participant recruitment

The sample was selected from mothers who had participated in the BiB COVID-19 surveys [11]. A purposive sample (n = 25) was taken to ensure the recruitment of British Muslim Pakistani women only, both English and Urdu speaking, with children of varying ages. Sampling also included women from different socio-economic backgrounds (based on the index of multiple deprivation). Participants were not selected on the basis of their reported symptoms of depression or anxiety in the BiB Covid-19 surveys as this information was provided to the research team in confidence. The purpose of the study was explained to non-English speaking women in Urdu by the bilingual researcher (HI), Participants were also offered the option of the interview being conducted in English or Urdu. Participants were recruited to the study between 20^th^ April– 30^th^ May 2022.

### Consent

The participant information sheet and consent form used in the BiB COVID-19 study did not contain a statement regarding participants’ agreement to be contacted for the study reported in this paper. Participants, however, had previously consented to be a part of BiB and for their research and routine health data to be used for research. An information pack containing a cover letter, information sheet and a useful contacts list of mental wellbeing support was sent, by post, to potential participants informing them about the study. After two weeks, the research team contacted the women again by phone and discussed with them what would be involved should they agree to participate. A date and time were arranged for those interested in participating. Potential participants who expressed an interest in taking part were given the option of participating by phone, via an online video platform, or in person. Prior to the interviews, participants were asked to read the information sheet. For the interviews, verbal consent was taken with participants and recorded over the phone at the beginning of the interview and logged into a study management spreadsheet. Authors had access to information that could identify individual participants during and after data collection.

### Reflexivity note

It is important at this juncture to discuss authors’ positionality to ensure transparency, credibility, and ethical conduct in the research process. It was crucial that the researchers questioned their own assumptions as we played an integral role in data collection and interpretation. HI and SI hold insider status as Urdu speaking, Muslim, British Pakistani women. HI, a qualitative researcher, resides in Bradford. Her research is on health inequalities faced by South Asian, Muslim women. SI, a qualitative researcher, conducted her PhD research with the Pakistani community in Bradford and has a special interest in the mental health of this population. BL and JD are British White women residing in Bradford. BL is a qualitative researcher with a special interest in gender and health inequalities. JD, a mixed methods researcher has a special interest in minority ethnic health and health inequalities. Our different identities and previous knowledge allowed us to unpick the data and question one another’s interpretations.

### Data collection

One-on-one interviews were deemed to be the most suitable data collection tool for the purpose of this study to enable detailed exploration of personal lived experiences [19] of participants during the pandemic. An interview guide was developed by HI and BL and included semi-structured, open-ended questions to facilitate discussions. Input was obtained from a researcher with experience of conducting interviews on the topic of mental health with Pakistani women. The guide was piloted on three British Muslim Pakistani women with family responsibilities living in Bradford, over the telephone and one in person and was refined based on their feedback. The data generated from the pilot interview is not included in this paper. The interview guide is displayed in Table 1. Participants were offered a £20 shopping voucher to thank them for taking part. Data were collected by HI through phone calls from April-May 2022 as per participants’ requests, at a time that suited them. For non-English speaking interviews, the interview questions were translated into Urdu while staying as close to their English meaning as possible to limit bias. Interview length averaged 55 minutes. Data collection continued until saturation i.e., no new insights emerged from the data [20]. Data saturation was reached by interview 25.

**Table 1:** Interview guide.

1. How has life been since the first lockdown in March 2020 when the first lockdown began? *(Prompt to think about what has changed/stayed the same)* 2. In what ways did the lockdowns impact on your day-to-day life? 3. What impact has this had on you mentally? *(prompt to think of mental and physical health)* 4. What is the difference in your day-to-day living as lockdown measures eased? 5. How would you describe your social life since the first lockdown? *(Prompt to think about what it was like before lockdown/how do you feel about this*?*)* 6. What do you feel helped stopped you from feeling down/negative or worrying excessively? *(probe for finances*, *religion*, *household structure*, *number and ages of children*, *feeling valued*, *respected*, *and supported)* 7. Finally, is there anything we haven’t talked about that you think we should know?

### Data analysis

Interviews were audio-recorded and transcribed by an NHS approved external transcription company. Five of the recordings were in Urdu and were translated and then transcribed by HI. The meanings of the words were translated, rather than re-writing the words, as is recommended when translating interviews [21]. Transcripts were reviewed for accuracy and anonymised by HI prior to importing into NVivo 12 qualitative analysis software to assist in the coding of data. The translated transcripts and the Urdu terms used in all transcripts were verified as being accurately translated by SI, who is also Urdu speaking alongside HI. Data were analysed using inductive thematic analysis as outlined by Braun and Clarke [22]. Line by line coding of 25 transcripts was undertaken by HI during ongoing data collection to develop an initial coding scheme. A codebook was then created and subsequently refined several times throughout the process by HI and BL. All authors worked together to create themes from the codes. Three representative transcripts were closed-coded by SI and BL to strengthen credibility and trustworthiness.

## Results

A total of 25 British Muslim Pakistani women with family responsibilities took part in the study. Twenty were English speaking and five spoke Urdu only. The number of people living within households ranged from 3 to 14. To protect participants identities, a range has been given rather than specific numbers of people per household. Participants lived in neighbourhoods with varying levels of deprivation. Table 2 displays participant characteristics. Pseudonyms are given to protect participant identities. A tick in the column means *yes* and an X in the column means *no*

**Table 2:** Participant characteristics.

Name	English speaking?	No of people per household	Living in 10% most deprived ward
Saira	✓	2–5	X
Shazia	X	6–10	X
Nasreen	✓	6–10	✓
Zuleikha	✓	2–5	X
Sana	✓	6–10	✓
Rifat	✓	10+	✓
Shamim	✓	6–10	X
Sobia	✓	6–10	X
Nilam	✓	6–10	✓
Qulsoom	✓	6–10	X
Nusret	X	6–10	X
Ruksana	✓	10+	✓
Sophia	✓	2–5	✓
Saba	✓	6–10	X
Aisha	✓	2–5	X
Tameena	✓	6–10	✓
Nyla	✓	2–5	✓
Zenab	X	6–10	X
Salma	✓	2–5	X
Fatima	X	6–10	✓
Iqra	✓	6–10	X
Rashida	✓	6–10	X
Taiba	✓	2–5	✓
Mariyah	✓	2–5	✓
Tazeem	X	6–10	✓

There were three themes identified in the data that demonstrated the impact of the COVID-19 pandemic on the lives of British Muslim Pakistani women with family responsibilities. The themes were (1) Community, cultural and religious contributors to poor mental wellbeing, (2) religious and cultural mediators of psychological distress, and (3), positive impacts on lifestyle. The themes and codes are displayed in Table 3 and are presented below with illustrative quotes. Quotes have been pseudonymised for purposes of anonymity.

**Table 3:** Themes and codes.

Theme: Community, cultural and religious contributors to poor mental wellbeing	Theme: Religious and cultural mediators of mental distress	Theme: Positive impacts on lifestyle
Code: • Community health anxiety • Reduction in religious and social interaction • Increased domestic load	Code: • Islamic religious faith • Household structure • Neighbours	Code: • Closeness to family • Closeness to faith • Work/life harmony

### Theme 1: Community, cultural and religious contributors to poor mental wellbeing

#### Community health anxiety

The findings present experiences that were described by British Muslim Pakistani women with family responsibilities affecting their wellbeing during the pandemic. Participants described fear and anxiety around catching the virus themselves. This concern was especially pronounced among participants considered clinically vulnerable. More than this though, many participants expressed worry at the prospect of their clinically vulnerable relatives getting the virus, and the potential implications this would involve:

“*I was so scared of one of my family getting it*. *My mother-in-law lives with us has cancer so we were very worried that one of us would give it to her and that she would die*. *The kids weren’t being as careful as they should have been and I was convinced they’d get it from outside and give it to her*.*”* (Rifat)

Some participants, especially those who were born and raised in Pakistan, expressed worry for relatives overseas, and hopelessness due to the distance between them:

“*I have my mum and siblings living in [city in Pakistan mentioned] and it was really bad there*. *So many people I grew up with*, *died*. *Every time I rang home*, *they’d tell me about how high the rates were there and that hospitals were full*. *I was so worried about being so far from them*. *What if something happened*?*”* (Shazia, Urdu speaking)

A key source of anxiety was the threat of high COVID-19 mortality and infection rates within the South Asian population. This was particularly worrying for some participants, as they feared they would contract the virus due to their community connectedness:

“*Everyone was talking about lots of deaths in ethnic communities*. *Like our Asians were dropping like flies really*. *Like Birmingham and Bradford where loads of our people live*. *We kept hearing of people in our community drying like we heard*, *“oh*, *so and so’s passed away*, *so and so’s passed away*.*” And obviously you’re part of that community*. *So it was a really worrying time*.*”* (Zuleikha)

#### Reduction in religious and social restriction

It was clear that social interaction was very important for participants and the inability to meet with others negatively affected their mental state. Participants explained how restrictions affected fundamental human interactions which were integral to their family, community, social and religious life. Ramadan took place one month after the first lockdown and participants reflected on how not being able to interact with others during the month made them feel:

“*You know like Ramadan came and normally*, *we go to the mosque for tarawee (special Ramadan prayers) and go to dawats (meals hosted by others) at people homes and make food for the mosque but we couldn’t do any of that*. *It was so isolating*, *and it made me very upset*. *It ruined Ramadan for me this year just because no one was together and that’s normally such a big part of Ramadan”* (Tameena)

Other participants mentioned the overwhelmingly large number of deaths in the Muslim community and not being able to attend funerals to pay their respects in person and participate fully in religious rituals and cultural traditions as normal:

“*When Muslims die*, *we do ghusl (ritual cleanse) on the body and shroud it*. *You know we go to the passed away persons house for three days to pay our respects and be there for the family but we couldn’t do any of that It was just all from a distance*. *Ring them*. *It was really bad and made me feel hopeless*. (Nasreen)

For some participants, not being able to visit relatives and friends for fear of giving or contracting the virus themselves, caused them sadness and had a long-lasting impact:

“*I couldn’t visit my family*. *My brother had come from Pakistan just before the lockdown and I didn’t see him for over a year*. *He’s the only family in England I have but I couldn’t go see him because I was so scared of getting covid or giving it to him*. *I still haven’t seen him*. *It makes me want to cry when I think about it”* (Nusret, Urdu speaking)

Others described interacting with others as part of their employment as a coping mechanism when they were feeling low due to imposed restrictions:

“*My job is in a school and being around people is what makes me happy and keeps my stress levels contained*. *During lockdown*, *my school closed so I was having to work from home so*, *I couldn’t talk or see people*. *My anxiety sky-rocketed because my coping strategy was that interaction with others”* (Nyla)

Although imposed restrictions had eased at the point of interview, the restrictions had lasting implications despite things returning to normal. For instance, some participants described feeling a lack of social connection with others:

“*The way we used to visit people’s houses or see friends before COVID has completely changed*. *Even though things are going back to normal*, *people are still keeping you away*. *Weddings are small gatherings now and no one calls you over like they used to do*. *Even with funerals*, *people still don’t want you to come over*. *I feel very disconnected*” (Sophia)

#### Increased domestic load

During the lockdowns, workplaces, schools, madrasas and extracurricular programmes ceased to operate. Nearly all participants noted the increase in domestic load during this period and the pressure they felt as a result:

“*Don’t even ask*. *So much*, *I can’t even begin to describe it*. *I was cooking nonstop*, *for the kids*. *They wouldn’t eat from out because of germs so I was constantly making food*. *Sometimes they would help me but it was too much and very stressful time”* (Sana)

Additional caring responsibilities of family members who were clinically vulnerable, and changes in family members ways of working were reported as challenging aspects and increased the difficulty of the pandemic

“*My anxiety was really worse in lockdown because I had to look after my father-in-law full time because he has kidney problems and I had no help from my husband because he was still working*. *My mother-in-law was depressed at that time too and it was very hard for me to look after her on top of looking after my father-in-law”* (Nilam).

Furthermore, feelings of resentment surfaced, and tensions were reported due to the lack of additional help received from family members. Participants perceived challenges in general expressions of wellbeing support from family members such as their husband:

“*My husband wasn’t working but didn’t help me out at all*. *What can I tell you about my husband*? *He’s a typical Asian man and doesn’t do anything at home*. *It caused so many arguments during that time”* (Aisha)

### Theme 2: Religious and cultural mediators of mental distress

#### Islamic religious faith

Whilst many participants discussed the mental burden imposed by COVID-19 and the impact of restrictions placed upon them, nearly all participants spoke extensively about how their religious beliefs and teachings were mitigating the negative effects of the pandemic on their mental wellbeing. These religious beliefs and teachings could be seen to help participants cope during the pandemic in two ways: belief in predestination and coping through prayer and supplication. Participants mentioned that their belief in Allah’s decree meant that despite how bad things were, this belief acted as a protective mechanism and consoled them:

“*The pandemic was a reminder that everything comes from Allah*. *The lockdown and COVID situation was a reminder to me that no matter what we think or do*, *death is inevitable and comes to everyone*. *That bought me a lot of comfort during the pandemic*.*”* (Nusret, Urdu speaking)

Participants described coping with the pandemic by praying regularly and supplicating, which gave them the strength and a sense of peace during uncertain times:

“*lockdown in one way it was like*, *you know*, *stressful at the beginning but it was our religious readings and namaz (prayer) that brought more peace to ourselves*.*”* (Rifat)

Some participants discussed the importance of being patient and expressing gratitude as part of their religious faith. This had a calming effect on them when experiencing difficulties during the pandemic. Examples were given of prophets and what they endured, which made the participants express gratitude when feeling negative:

“*there’s always light at the end of the tunnel and it’s all the knowledge that you’ve sort of accumulated about the deen and you know*, *why sometimes we can feel down and it’s normal to feel down but*, *you know*, *and you look at the lives of the Prophet Muhammad*, *peace be upon him and the other prophets and then you think*, *well actually*, *mine’s not so bad*, *so it kind of keeps you going*.” (Shamim)

#### Household structure

Larger household structures appeared to serve as a protective factor for some participants. Although this meant that the domestic load was greater, it also meant that often their children could share the housework chores, thus lessening the burden on the participant:

“*My daughters helped me a lot with cooking*, *cleaning and laundry*, *They could see I had so much to do and they stepped in*. *My sons would do what they could with that too but they helped in other ways around the house*.*”* (Taiba)

Some participants lived with extended family which included in-laws, aunts and uncles. This large household was described as being useful for sharing the domestic load:

“*big family*, *me and my sister-in-law all day when breakfast finish*, *clean everywhere*, *then all dinner time*, *then dinner time finish*, *oh evening teatime*, *this is thing too much normally for people but more people in house to help in my family*.*”* (Ruksana)

#### Neighbours

Participants stated that they lived in neighbourhoods that were populated with predominantly Muslim, Pakistani families. Islamic religion and Pakistani culture both encourage the good treatment of neighbours, which includes generosity. Participants described the generosity of neighbours sending meals, checking in on them and offering support as being a source of great help during the pandemic:

“*Our neighbours went shopping for us*, *they cooked for us*. *My next-door neighbour checking up on us*. *They helped us so much*. *I’m not going to lie*, *they said if we need anything at all*, *we will help in every way we can*. *That was really reassuring at that time*, *and helped reduce my tension”* (Sobia)

This was especially true for participants that were born and raised in Pakistan and did not have relatives close by or other means of support:

“*Our neighbours are all Pakistani and I don’t have family here but neighbours are so supportive and helped me so much during the pandemic and I’m really grateful for them*. *They fill the void I don’t have with my family not being here*.*”* (Fatima, Urdu speaking)

### Theme 3: Positive impacts on lifestyle

#### Closeness to family

Lastly, although there was considerable discussion on the negative aspects of the pandemic on mental wellbeing, participants also spoke extensively of the positive outcomes. For example, the restrictions on day-to-day life meant that people had to stay at home. This meant that there was more opportunity to spend time together as a family:

“*It’s the first time we could sit together and eat as a family*. *Life gets so busy and you don’t get time to enjoy spending time at home so it was nice to just chill with my kids and to pray together*. *My daughter has been wating to bake with me for ages but I’ve been too busy to do it before so it was so nice to bake with her”* (Mariyah)

Relationships between participants and estranged family members such as in-laws, had improved in lockdown. One participant noted the positive impact of the pandemic on her relationship with her husband:

“*my husband and I used to fight like cat and dog so we were living separately*. *COVID made us change how we see things*. *We realised life’s too short for this fighting*, *so I let him move back in with us and we’ve been getting along so much better*.*”* (Taiba)

Participants also noted that after the lockdowns had ended and life had returned to normal, their domestic load was now shared by other family members such as the husband and/or elder children whereas it was their sole responsibility pre-pandemic:

“*my husband and kids*, *bless them*, *they’ve continued to help out with housework*. *My eldest cooks a few times a week now*. *She started to do it in the first lockdown to help me out because it was too much but now it’s become routine”* (Shamim)

#### Closeness to faith

Participants also disclosed that having more time meant that they and their household members had increased their adherence to their religious faith during lockdown:

“*The best change was my kids start praying five times a day and reading Quran as well because they’re home I told them you’ve got the opportunity so don’t lose it*. *That’s a very good thing to happen and we’ve been reading religious texts*, *and when my husband was home*, *he’s been doing jamat (congregation in prayer) and we have been reading namaz (prayer) behind him so that’s a really good opportunity*. *I just told all my kids and my husband*, *I said we lost something but we gained something really good which is ours and nobody can take it away from us and our kids*.*”* (Sobia)

Ramadan began in April 2020, a month after the first lockdown. Participants described the peace they felt in the month, and how lockdown enabled them to fully engage in worship as a family:

“*my family*, *Alhamdulillah (paise be to God)*, *like you know we’ve been having a really*, *really good time and that time Ramadan come as well and really good timing like you know*, *so my kids have kept all their rozay and been reading the namaz and tarawee (voluntary night prayers in Ramadan) with jamaat*. *We were focused on Ramadan praying and that made us think a lot less about COVID so we were happier*. *Ramadan was just dedicated to ourselves”* (Sophia)

For some participants, this enabled the establishment of a routine around prayer two years on from Ramadan:

“*They were doing it before the lockdown but on and off*, *like you’re sometime lazy and miss it but in the lockdown they got into such a good routine mashallah (God willed it)*, *they’ve kept up with it*, *never even missing the mosque no matter what happens*, *they still keep up with it*. *Then on top of that they started to read Quran and have kept up with that too”* (Nasreen)

#### Work/life harmony

Finding work/life harmony was reported to be a struggle before the pandemic, according to participants. Once lockdown happened, the pace of life slowed down, and routines changed as a result. Suddenly, large portions of the day were no longer taken up by school runs, dropping off and picking up their children to mosque, or non-essential meetings. Participants enjoyed the slower pace, and noted the lack of stress:

“*I’ve noticed I’ve slowed down and I feel like before COVID*, *I used to always feel like I have to go somewhere*, *I have to be with the children*, *I have to be in the car*, *have to plan this trip*, *have to plan that trip*. *I just feel less pressure now*. *That’s one of the best things that’s come out of this”* (Salma)

Some Pakistani women spoke of how the restrictions relieved them of social obligations such as visits to friends and extended family. Participants expressed some of these relationships were generally detrimental to their wellbeing, and the understanding by others that frequent visitation was no longer possible had a positive impact:

“*when you have like extended family and some people are a little bit toxic and the fact that I wasn’t seeing them often was a bit of a blessing*. *They couldn’t say anything to me about it either*, *because of COVID”* (Saba)

## Discussion

The findings from this qualitative study exploring the mental wellbeing of British Muslim Pakistani women with family responsibilities during the COVID-19 pandemic found that mental health was particularly affected by the prospect of extended family living in the same house contracting the virus, as well as concern for relatives living abroad. Anxieties were exacerbated by the high virus transmission and deaths in the South Asian population. The lack of religious interaction during Ramadan and the inability to attend funeral rituals and participate in cultural traditions were an additional source of worry. Religious beliefs served as protective factors against poor mental wellbeing in Pakistani women, as well as support they received from their community networks and the family, particularly the support from family members which reduced household burdens and therefore allowed them respite. Positive outcomes of the pandemic were also highlighted such as spending more time with family, becoming more religiously observant and welcoming the slower pace of life.

Our findings show that Pakistani women were very concerned about family members contracting the virus. Although this has been identified as a worry among the general population in the wider literature [11, 23, 24], it was especially notable among Pakistani women in this study because many of them lived with extended, older family which included their mothers, aunts, and in-laws. Intergenerational living with larger household sizes are common among South Asians [25]. Research has shown that in individuals presenting with suspected COVID-19, those from ethnic minority communities and larger households had an increased likelihood of testing positive for the virus [26]. People from ethnic minority communities, therefore, may have intense fears and anxieties in relation to the increased risk of COVID-19 in their communities [27]. Like our study, other studies have also revealed a mental health impact from the lockdown on minority ethnic women with family responsibilities, with increased domestic load being cited as a reason for increased anxiety in these populations due to large household sizes [28]. Our study found that the lack of Pakistani husbands’ support with household chores exacerbated their stress levels. Our study, however, also found that extended family members living in the same household alleviated the tension by offering domestic support during lockdowns. It is important to note, however, that it was primarily female family members that provided domestic support, such as daughters and aunts. Often, the negative aspects of living in large households have been focused on but as our study shows, living in large households resulted in both positive and negative outcomes for women.

Physical distancing from social networks have been some of the most challenging lifestyle changes made by individuals during the COVID-19 pandemic [29]. Like other studies, we found that restrictions on interactions with others caused considerable distress [30]. For the Muslim community, however, there were specific consequences of restrictions on cultural and religious practices [24]. Practices such as coming together as a community to break the fast during Ramadan, and not being able to visit family for the Eid celebration, caused much distress to this population. A further example can be seen in funeral practices in this population. There are ethnic, cultural and religious differences in funeral practices [31] which were affected to varying degrees by the COVID-19 restrictions during the lockdowns. The ritual act of washing the body of the diseased, for example–a crucial act in Islam–was, at the time, being carried out wearing full personal protective equipment with supervision or not at all [32]. A ritualistic three days of Islamic mourning is observed by Muslims, wherein the community and family provide support to one another [33]. This religious mourning process was disrupted by COVID-19. As seen in this study, the impact of restrictions on funeral practices in the Pakistani Muslim community are a source of communal as well as individual distress. These cultural and religious practices are integral to the sense of belonging and connectedness within the Muslim community and the impact of restrictions on these practices have had continued effects even after restrictions were lifted. Evidence from our study and those of others recommend the need for further research to develop in-depth understanding of the role of culture and religion in populations’ coping during pandemics and the importance of considering culture and religion when developing COVID-19 guidance [24, 34].

Participants spoke of coping strategies to mitigate the psychological impact of the pandemic on their wellbeing. A range of coping strategies during the COVID-19 pandemic have been identified in the wider literature, which include engaging in hobbies and activities and staying connected with others [23]. In this study, however, women spoke extensively of religious coping mechanisms. For many people from minority ethnic backgrounds, religion can provide social support, a sense of meaning and connection, and more importantly, exposure to social norms linked with the group membership [35]. In our study, religion and spirituality enabled British Muslim Pakistani women with family responsibilities to cope and thus offered respite from the psychological distress associated with the COVID-19 pandemic. Central to this was the acceptance of the situation caused by the pandemic, expressions of gratitude and patience as propagated by prophets, and a belief in predestination. Positive religious coping is associated with improving physical and mental health outcomes, whereas negative religious coping has been linked with increased stress and anxiety [36]. Other studies have also found that positive religious coping provided individuals with a protective buffer against negative mental wellbeing in those reporting a strong religious identity during the COVID 19 pandemic [24, 37]. Positive religious coping, therefore, may help these populations reduce their risk of poor mental wellbeing, which could be of great benefit for Muslim communities more generally across the UK. An exploration of religious coping may prove beneficial in informing broader national pandemic preparedness plans in the future.

Our study found that support from neighbours served as a protective factor, especially among women who were born and raised in Pakistan and did not have extended family in England. Many of the women in our study reported residency in areas of high own group density. Areas of high ethnic density have been shown to buffer against the effects of severe mental illness in South Asians relative to White British populations with severe illness [38]. Community assets such as community cohesion and neighbourliness within a community can support health and wellbeing [39]. It has been previously reported that in deprived areas of Bradford, Pakistani women are more likely to report low family social capital compared to White British women [40]. Our findings indicate that neighbourhood relations might become more important in times of societal crisis and social restriction measures, when public support services may decrease, or support from the family is limited. Findings from this study indicate that neighbourhoods with high social capital seem to be more resilient to crisis like the COVID-19 pandemic [41]. This corresponds with findings from previous research, suggesting that a high level of social capital promotes health [42, 43], and may help to explain why women from Pakistani and Bangladeshi ethnic minority backgrounds had greater prevalence of depression in London than in Bradford. A more thorough understanding is needed of effective strategies for building and maintaining social capital during future crises as social capital may increase adherence to physical distancing and other protective behaviours in populations.

Throughout the COVID-19 pandemic, efforts were made to incorporate learning from previous epidemics in the development of wellbeing guidelines and support interventions, but many of these guidelines and interventions focused mainly on the assessment of clinical outcomes, which often excluded cultural descriptions of wellbeing and mental health [44]. In terms of coping with the damaging mental health effects of the COVID 19 pandemic, this study found that British Pakistani women relied on informal support from family and friends. Dong et al. (2022) found that addressing cultural factors and support mechanisms would improve mental health and wellbeing [45]. Also, some British Pakistani women stated that they used their own methods to cope, such as religion and spirituality. This supports growing evidence that religious coping improved wellbeing [46]. Enhancing mental health and wellbeing through spiritual and faith-based groups will equip British Pakistani women with resilience building tools, self-care and coping strategies, and empower them to seek support when needed. Our study provided insight into excessive responsibilities of British Pakistani women that prevented them from carrying out their roles as they would have liked, particularly in the case of their responsibilities towards loved ones. Research has found the fear and anxiety of passing on COVID-19 to family members is a persistent concern and for British Pakistani women, impacted their descriptions of wellbeing. This emphasised the need to include this in pandemic response guidelines. Furthermore, it appears that social support is an integral element of maintaining British Pakistani women’s mental health as previous studies have found that a lack of social support affects anxiety, and stress [47]. It is important to foster supportive networks in future pandemics, through encouraging peer support and sharing of experiences to mitigate the negative impact on mental health.

This study includes in-depth experiences of British Muslim Pakistani women, a community who are considered ‘seldom-heard’ and ‘under-researched’ and by including their perspectives in this research, we were able to understand both mental health risk and protective factors in this population that differed from other communities. We also included non-English speaking women whereas often, non-English speakers are excluded from research due to language barriers [48]. A key strength of this study is that the researcher (HI) held insider status as a British Muslim Pakistani mother living in Bradford. Research is often conducted by outsiders to communities without specific and local knowledge, which could result in, among other issues, participants’ mistrust of researchers, limited cultural insights by researchers, and lack of acceptance by the community [49, 50]. The insider position held by HI was important for facilitating discussion and enabled participants to express their experiences in ways and via language universally familiar within the both the Muslim and the Pakistani community.

It is possible that the insider status of HI could have resulted in bias, given her knowledge, and understanding of the community [51]. This issue was mitigated by HI being extra vigilant about her line of questioning and by allowing the interview to flow naturally with her limited input. Another limitation is that participants may have omitted details under the assumption that HI had insider knowledge. This issue was managed by HI asking participants for clarification when they were being vague. By focussing on British, Muslim, Pakistani women with family responsibilities living in the city of Bradford, some of the findings may be specific to that population. Future research would benefit from obtaining perspectives from a wider demographic. In addition, as data were not collected on whether participants were living with a diagnosed mental health condition, findings cannot be generalised to Pakistani Muslim women who have a history of mental health disorders as their experiences during the COVID-19 pandemic may have differed to the women in our study.

## Conclusion

This study found that British Muslim Pakistani women with family responsibilities living in the UK were psychologically distressed by the high rates of virus transmission in their ethnic community and at the prospect of older members of their extended family, who lived with them, developing the virus. The impact of restrictions on fundamental religious and cultural interactions further exacerbated poor mental wellbeing in this population. Religion, community social capital and larger household structures were all protective factors for British Muslim Pakistani women with family responsibilities. An exploration of religious and cultural coping mechanisms should be used to inform future national pandemic preparedness plans, as well as effective strategies for building and maintaining social capital. This may increase adherence to physical distancing and other protective behaviours in populations. Attention should also be given to ways in which social capital and protective factors within communities could be best utilised for mental health policy and intervention.
